# Association of Smoking With Semen Quality and µ-Calpain Level in Normospermia: A Case-Control Study 

**Published:** 2016-03

**Authors:** Damoon Ashtary-Larky, Mohammad Ali Ghaffari, Mozhgan Noorbehbahani, Meysam Alipour

**Affiliations:** 1 Student Research Committee, Ahvaz Jundishapur University of Medical Sciences, Ahvaz, Iran; 2 Cellular and Molecular Research Center, Ahvaz Jundishapur University of Medical Sciences, Ahvaz, Iran; 3 Department of Clinical Biochemistry, School of Medicine, Ahvaz Jundishapur University of Medical Sciences, Ahvaz, Iran; 4 Nutrition and Metabolic Diseases Research Center, Ahvaz Jundishapur University of Medical Sciences, Ahvaz, Iran

**Keywords:** Case-Control Study, Smoking, Semen, Calpain

## Abstract

**Objective:** Calpains are a family of Ca^2+^ dependent proteases. There is some evidence that calpains involved in fusion process that occurs between spermatozoa and the oocyte. The current study aimed to investigate the association of smoking with semen quality and µ-calpain level.

**Materials and methods:** This case-control study was conducted on 117 normospermia males between June 2013 and march 2014 in Jahad Laboratory in ahvaz, Iran. The semen samples were collected from male smokers (n = 50) and non-smokers (n = 67). We divided these participants as light, moderate, or heavy smokers based on their cigarettes per day (CPD). ELISA assays were used to measure µ-calpain concentration. All semen samples were analyzed according to World Health Organization guidelines.

**Results:** The analysis of semen showed the volume, concentration, motility and morphology of semen were significantly lower among the smoker men than the non-smoker men. Also this significant difference was observed based on the number (light, moderate and heavy smokers) and duration (short term and long term smoker) of smoking. Although, showed no significant difference between µ-calpain of smoker men and non-smoker men. CPD showed negatively correlation with semen volume, concentration, motility and morphology of sperm.

**Conclusion:** Sperm quality was negatively correlated with CPD and duration of smoking. However, there is no significant correlation between smoking and µ-calpain concentration.

## Introduction

Infertility has been known as a critical public health problem globally. Based on World Health Organization (WHO) definition, Infertility is “a disease of the reproductive system defined by the failure to achieve a clinical pregnancy after 12 months or more of regular unprotected sexual intercourse (1). Roughly 15% of couples are infertile, and among these, approximately 50% of causes are due to male infertility (2). Cigarette smoking as a health issue is a major cause of mortality owing to lung cancer and cardiovascular disease. Previous studies have demonstrated that approximately one-third of the world’s populations over the age of 15 years are smokers (3, 4). Due to the high population of male smokers worldwide, and the fact that cigarette smoke contains known mutagens and carcinogens, there has been much concern that cigarette consumption can have adverse effects on male reproduction (5).

Calpains are a family of Ca^2+^ dependent thiol proteases that are widely expressed in mammalian cells. Calpains exist in both ubiquitous and tissue specific isoforms (6). These proteins are involved in various calcium-regulated cellular mechanisms including remodeling of cytoskeletal/membrane attachments, different signal transduction pathways, membrane fusion, platelet activation, cell cycle progression and apoptosis (7, 8). Previous studies have suggested that calcium plays an important role in biochemical processes that enable spermatozoa to penetrate the oocyte. One of the processes which calpains are involved is the membrane fusion process that occurs between the plasma membranes of the spermatozoa and the oocyte (9). In mammalian sperm, µ-calpain and m-calpain take place in the acrosomal cap; Inducing capacitated spermatozoa to undergo the acrosome reaction. A selective calpain inhibitor can decrease the acrosome reaction rate in a dose-dependent manner (10-12). µ-calpain is relocated and translocated from cytoplasm to plasma membrane during capacitation where it could cleave spectrin, one of the proteins of the plasma membrane-associated cytoskeleton, and facilitates acrosome reaction (13).

Cigarette smoking can have profound effects on cell process in spermatozoa but the mechanisms of cigarette smoke–associated damage to human spermatozoa are still largely unknown. Therefore, the present study was designed to investigate the association between cigarette smoking and sperm quality and µ-calpain concentration in smoker normospermia male.

## Materials and methods

***Study population***

This was a case-control study performed in Ahvaz, Iran, between June 2013 and march 2014. Study population was selected from normospermia males who attended Jahad Laboratory for routine semen analyses. We studied seminal fluid of 117 men from a population of 213 volunteers. Of those, 67 were nonsmoker (control), 50 were smoker (case). Men who were ex-smokers, hookah smokers or with a history of addiction, recreational drug use or alcohol consumption, having fever, occupational exposure to chemicals, gonad toxins and toxic heavy metals or excessive heat, varicocele or other anatomical injury, leukocytospermia or chronic genitourinary infections diagnosed by spermiocultures, altered hormonal levels, were excluded from this study. 

History of tobacco consumption and other lifestyle patterns were noted. we divided case group into three subgroups based on their cigarettes per day (CPD) and duration of smoking. People were classified as a non-smoker, light, moderate and heavy smoker. For the purposes of the study, a person was classified as a smoker if they smoked at least 1 CPD for at least 12 months. Heavy smokers were classified as those who smoked, on average, more than 20 CPD, moderate smokers as 11-20 CPD, and light smokers as 10 or less than 10 CPD (3). Moreover, subjects categorized as a short-time smoker (1-10 years, n = 39) and log-term smoker (11-20 years, n = 11) (14). 

***Semen samples***

The semen samples were collected in a private room in the laboratory after a minimum of 2 days and a maximum of 7 days of sexual abstinence. The specimen container kept at ambient temperature at 37 ^°C^, to avoid large changes in temperature that may affect the spermatozoa after ejaculation and processed immediately after complete liquefaction (15). All semen samples were analyzed for volume, PH, sperm motility, sperm concentration, and sperm morphology according to WHO guidelines (15). An informed consent form was gained from subjects and the study protocol was approved by the ethics committee of the Ahvaz Jundishapur University of Medical Sciences.

***µ-calpain isolation from sperm cells***

µ-calpain of each semen sample was partially isolated as previously described by Lee et al. (16). Briefly, human fresh semen (1 ml) was centrifuged at 1000 × g for 10 minutes (Eppendorf AG, Hamburg, Germany). Then the pellets were suspended in 1 ml phosphate buffered saline (PBS) and sonicated by ultrasonicator for 1 minute at 10-s intervals (Hielscher UP50H ultrasonic, Germany). Centrifugation of extracts was performed for 20 minutes at 27000 × g (Zentrifugen, Micro 220R, Hettich, Germany). The precipitates were washed once with 1 ml PBS followed by resuspension and centrifugation as mentioned above. The supernatant of samples were stored at -80^˚C^ until assayed.

***Determination of µ-calpain concentration***

Concentration of µ-alpain within each sample was measured by commercial ELISA kit (Eastbiopharm, China). The method uses a double-antibody sandwich enzyme-linked immunosorbent assay (ELISA) to assay the level of human µ-calpain. 

***Statistical analysis***

Results are presented as mean ± standard deviation. Experiments of semen parameters were done in triplicate and the mean was calculated for each subject. Normal distribution of quantitative data was measured by the Kolmogorov-Smirnov test. To compare differences between the case and control groups, Independent-sample t-test for normally distributed variables and Mann-Whitney U test for non-normally distributed variables were used. To compare differences between the subgroups, Tukey test for normally distributed variables and kruskal wallis test for non-normally distributed variables were used. Pearson correlation coefficient was calculated with the use of SPSS (version 19). p ≤ 0.05 was considered statistically significant.

## Results

Table 1 presents baseline characteristics of 50 (42.7%) smokers as the case and 67 (57.3%) non-smoker as the control groups. The case and control groups matched in age. The average age of the men was 26.4 ± 4.8 years. Case group started smoking within at least previous 12 months.

**Table 1:** Baseline characteristics of the case and control groups

	**Case ** **group**	**Control ** **group**	**p value**
Number [n (%)]	50 (42.7)	67 (57.3)	-
Age (year)	26.9± 4.9	26.0± 4.7	NS
CPD	13.4± 10.9	0	< 0.05^a^

*NS: non-significant; CPD: Cigarettes per day;

a Significant difference between groups (p < 0.05)

Table 2 shows the volume, concentration, motility and morphology of semen were significantly lower among the smoker men than the non-smoker men. Although, shown no significant difference between PH and µ-calpain level of smoker men and non-smoker men.

Table 3 indicate the significant difference in volume, concentration, motility and morphology of semen between case subgroups based on duration of smoking (short term and long term smokers ) with control group. 

**Table 2:** Comparison of variables between case and control groups

	**Case group**	**Control group**	**p value**
Semen volume (ml)	2.3 ± 0.7	3.20 ± 0.60	< 0.05 a
PH	8.02 ± 0.1	8.08 ± 0.10	NS
Concentration × 10⁶	49.4 ± 7.3	62.66± 9.60	< 0.05 a
Motility (%)	44.5 ± 6.3	53.20 ± 8.40	< 0.05 a
Normal morphology (%)	29.4 ± 8.0	39.20 ± 13.2	< 0.05 a
µ-Calpain (ng/ml)	25.28 ± 1.0	26.88 ± 1.30	NS

NS: non-significant;

a Significant difference between groups

**Table 3:** Comparison of variables between case and control groups based on duration of smoking

	**Case group**		**Control group**	**p value**
	**Short-term**	**Long-term**		
Semen volume (ml)	2.40 ± 0.7	1.9 ± 0.7	3.20 ± 0.6	< 0.05^a^,^b^
PH	8.04 ± 0.1	7.96 ± 0.1	8.08 ± 0.1	NS
Concentration × 10⁶	50.56 ± 7.3	44.64 ± 5.2	62.66 ± 9.6	< 0.05 a,b
Motility (%)	45.40 ± 6.1	44.90 ± 5.1	53.20 ± 8.4	< 0.05 a,b
Normal morphology (%)	31.90 ± 7.10	20.60 ± 4.0	39.20 ± 13.2	< 0.05 a,b
µ-Calpain (ng/ml)	25.75 ± 7.2	23.58 ± 7.3	26.88 ± 1.3	NS

NS: non-significant;

a Significant difference between short-term smokers compared with non-smokers;

b Significant difference between short-term smokers compared with non-smokers

**Figure 1:**
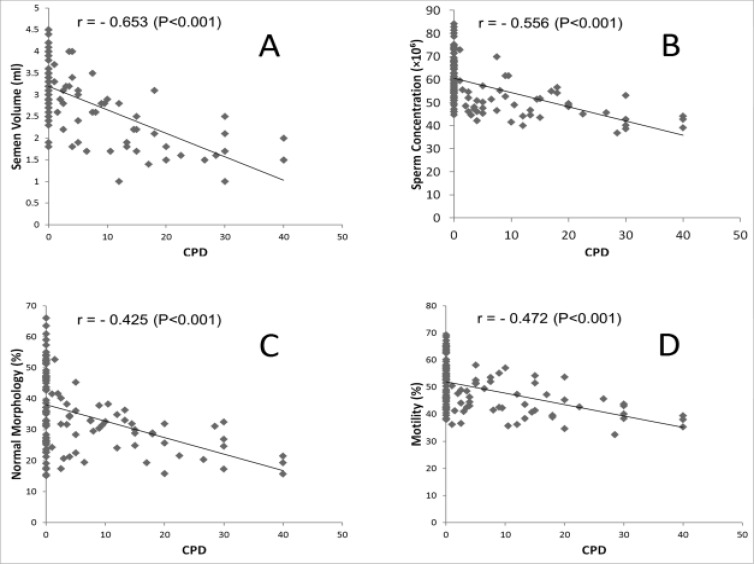
The relationship between CPD and semen volume (A), sperm concentration (B), percentage of normal morphology (C) and sperm motility (D)

Table 4 compared study variables in three subgroups of smoker according to number of smoking in day (Light, moderate and heavy smokers) with control group. Table 4 showed volume, concentration, motility and morphology of semen were significantly lower among subgroups of cases than the control group. Also, shown no significant difference in µ-calpain level between groups. CPD showed negatively correlation with semen volume (r = -0.0653, p < 0.001), concentration(r = -0.0546, p < 0.001), motility (r = -0.0425, p < 0.001) and percentage of normal morphology (r = -0.0472, p < 0.001) of sperm (Figure 1).

**Table 4:** Comparison of variables between case and control groups based on cigarettes per day

	**Case group**			**Control group**	**p value**
	**Light**	**Moderate**	**Heavy**		
Semen volume (ml)	2.80 ± 0.6	1.90 ± 0.5	1.70 ± 0.4	3.20 ± 0.6	< 0.05^a^,^b^,^c^
PH	8.08 ± 0.13	8.03 ± 0.2	8.03 ± 0.2	8.08 ± 0.1	NS
Concentration × 10⁶	52.30 ± 7.9	49.00 ± 4.6	42.80 ± 4.6	62.66 ± 9.6	< 0.05^a^,^b^,^c^
Motility (%)	47.60 ± 6.3	43.20 ± 6.4	39.9 ± 4.0	53.20 ± 8.4	< 0.05^a^,^b^,^c^
Normal morphology (%)	32.40 ± 7.10	28.80 ± 6.1	23.1 ± 5.6	39.20 ± 13.2	< 0.05^a^,^b^,^c^
µ-Calpain (ng/ml)	25.35 ± 1.5	25.21 ± 2.0	25.18 ± 2.0	26.88 ± 1.3	NS

NS: non-significant;

a Significant difference between Light smokers compared with non-smokers;

b Significant difference between Moderate smokers compared with non-smokers;

c Significant difference between Heavy smokers compared with non-smokers

## Discussion

Many studies reported negatively correlation between cigarette smoking and semen quality (17-19). Calpain in sperm is a component of the biochemical processes that regulate the fertilizing capacity of human spermatozoa (20). The effect of cigarette smoke on concentration of µ-calpain in human sperm cells is understood. 

In the present study, we have observed a statistically significant relationship between cigarette smoking and several semen characteristics. The results support the fact that smoking has an adverse effect on human semen quality as previously mentioned (21-23). To better understand the negative effects of CPD on the semen quality, we divided the smoker subjects into 3 groups (mild, moderate, and heavy smokers) and compared them with the non-smoker group. Based on our results, some parameters of sperm quality strongly depend on smoking, CPD and duration of smoking. The results of our research showed significant differences in semen volume, sperm concentration, motility and percentage of normal morphology in smokers compared with non-smokers. On the other hand, a comparison of calpain from different groups of smoker and nonsmokers did not reveal any significant difference.

Duration of smoking could affect the quality of sperms (24). The current study finding indicated significant differences between long-term smokers, short-term smokers and non-smokers. The findings of the present study consistent with numerous other studies, which show a significant relation between smoking duration and sperm concentration, as well as between the number of cigarette smoked daily, sperm concentration and motility (14, 25). Moreover, No dose dependence of CPD and duration of smoking on calpain levels was seen in our study. The present study demonstrated that CPD and duration of smoking were correlated with semen volume, sperm concentration, percentage of normal morphology and sperm motility. Furthermore, duration of smoking can effect on PH of seminal fluid. Our finding showed that there were no significant correlation between µ-calpain and CPD, duration of smoking and all seminal characteristics. 

These results support those studies which report the negative effect of cigarette smoking on sperm quality (26, 27). The mechanisms of the effect of smoking on sperm characteristics are still not completely understood. Cigarette has about 4000 chemical components like carbon monoxide, nitrogen oxide, hydrocarbons, tar, hydrogen cyanide, nicotine, etc. About 40 type on these components have are carcinogen (28). These chemical components, especially nicotine adversely affect semen quality (29, 30). It seems that level of µ-calpain in spermatozoa does not depend on cigarette consumption. More researches are needed to determine the effect of cigarette smoking on activity of µ-calpain and its gene expression.

We found the adverse effect of smoking on sperm quality according to number and duration.

Although the µ-calpain level was not affected by smoking, but smokers show significantly lower sperm quality. Further studies are needed to determine Impact of smoking on µ-calpain and its mechanisms.

## References

[1] Zegers-Hochschild F, Adamson GD, de Mouzon J, Ishihara O, Mansour R, Nygren K (2009). "The international committee for monitoring assisted reproductive technology (ICMART) and the world health organization (WHO) revised glossary on ART terminology. Hum Reprod.

[2] Kooti W, Mansouri E, Ghasemiboroon M, Harizi M, Ashtary-Larky D, Afrisham R (2014). The Effects of hydroalcoholic extract of apium graveolens leaf on the number of sexual cells and testicular structure in rat. Jundishapur J Nat Pharm Prod.

[3] Ghaffari MA, Rostami M (2013). The effect of cigarette smoking on human sperm creatine kinase activity: as an ATP buffering system in sperm. Int J Fertil Steril.

[4] Pope CA, Burnett RT, Turner MC, Cohen A, Krewski D, Jerrett M (2011). Lung cancer and cardiovascular disease mortality associated with ambient air pollution and cigarette smoke: shape of the exposure-response relationships. Environ Health Perspect.

[5] Gaur DS, Talekar M, Pathak VP (2007). Effect of cigarette smoking on semen quality of infertile men. Singapore Med J.

[6] Perrin BJ, Huttenlocher A (2002). Calpain. Int J Biochem Cell Biol.

[7] Goll DE, Thompson VF, Li H, Wei W, Cong J (2003). The calpain system. Physiol Rev.

[8] Suzuki K, Hata S, Kawabata Y, Sorimachi H (2004). Structure, activation, and biology of calpain. Diabetes.

[9] Rojas FJ, Brush M, Moretti-Rojas I (1999). Calpain-calpastatin: a novel, complete calcium-dependent protease system in human spermatozoa. Mol Hum Reprod.

[10] Yoneda R, Takahashi T, Matsui H, Takano N, Hasebe Y, Ogiwara K (2013). Three testis-specific paralogous serine proteases play different roles in murine spermatogenesis and are involved in germ cell survival during meiosis. Biol Reprod.

[11] Rojas FJ, Brush M, Moretti-Rojas I (1999). Calpain-calpastatin: a novel, complete calcium-dependent protease system in human spermatozoa. Mol Hum Reprod.

[12] Aoyama T, Ozaki Y, Aoki K, Kunimatsu M, Tada T, Sasaki M, Suzumori K (2001). Involvement of mu-calpain in human sperm capacitation for fertilization. Am J Reprod Immunol.

[13] Bastián Y, Roa-Espitia AL, Mújica A, Hernández-González EO (2010). Calpain modulates capacitation and acrosome reaction through cleavage of the spectrin cytoskeleton. Reproduction.

[14] Zhang JP, Meng QY, Wang Q, Zhang LJ, Mao YL, Sun ZX (2000). Effect of smoking on semen quality of infertile men in Shandong, China. Asian J Androl.

[15] World Health Organization (2010). WHO laboratory manual for the examination and processing of human semen.

[16] World Health Organization ( 2010). WHO Laboratory Manual for the Examination and Processing of Human Semen.

[17] Lee CY, Huang YS, Huang CH, Hu PC, Menge AC (1982). Monoclonal antibodies to human sperm antigens. J Reprod Immunol.

[18] Trummer H, Habermann H, Haas J, Pummer K (2002). The impact of cigarette smoking on human semen parameters and hormones. Hum Reprod.

[19] Collodel G, Capitani S, Pammolli A, Giannerini V, Geminiani M, Moretti E (2010). Semen quality of male idiopathic infertile smokers and nonsmokers: an ultrastructural study. J Androl.

[20] Hassa H, Yildirim A, Can C, Turgut M, Tanir HM, Senses T (2006). Effect of smoking on semen parameters of men attending an infertility clinic. Clin Exp Obstet Gynecol.

[21] Rojas FJ, Moretti-Rojas I (2000). Involvement of the calcium-specific protease, calpain, in the fertilizing capacity of human spermatozoa. Int J Androl.

[22] Künzle R, Mueller MD, Hänggi W, Birkhäuser MH, Drescher H, Bersinger NA (2003). Semen quality of male smokers and nonsmokers in infertile couples. Fertil Steril.

[23] Sepaniak S, Forges T, Gerard H, Foliguet B, Bene MC, Monnier-Barbarino P (2006). The influence of cigarette smoking on human sperm quality and DNA fragmentation. Toxicology.

[24] Gaur DS, Talekar M, Pathak VP (2007). Effect of cigarette smoking on semen quality of infertile men. Singapore Med J.

[25] Kidd SA, Eskenazi B, Wyrobek AJ (2001). Effects of male age on semen quality and fertility: a review of the literature. Fertil Steril.

[26] Ramlau-Hansen CH, Thulstrup AM, Aggerholm AS, Jensen MS, Toft G, Bonde JP (2007). Is smoking a risk factor for decreased semen quality? A cross-sectional analysis. Hum Reprod.

[27] Mostafa T (2010). Cigarette smoking and male infertility. Journal of Advanced Research.

[28] Meri ZB, Irshid IB, Migdadi M, Irshid AB, Mhanna SA (2013). Does cigarette smoking affect seminal fluid parameters? A comparative study. Oman Med J.

[29] Davar R, Sekhavat L, Naserzadeh N (2012). Semen parameters of non-infertile smoker and non-smoker men. J Med Life.

[30] Oyeyipo IP, Maartens PJ, du Plessis SS (2014). In vitro effects of nicotine on human spermatozoa. Andrologia.

[31] Joursaraei S GH A, Shibahara H, Ayustawati, Hirano Y, Shiraishi Y, Khalatbari A (2008). The in-vitro effects of nicotine, cotinine and leptin on sperm parameters analyzed by CASA system. Iranian Journal of Reproductive Medicine.

